# Illicit drug abuse and complexity of tibial shaft fracture based on AO/OTA classification: Is there any connection?

**DOI:** 10.1002/jeo2.12003

**Published:** 2024-01-23

**Authors:** Amirmohammad Sharafi, Ali Ghaderi, Parmida Shahbazi, Amirhossein Ghaseminejad‐Raeini, Akam Ramezani, Mohammad Soleimani, Parham Talebiyan, Seyyed Hossein Shafiei

**Affiliations:** ^1^ Orthopedic Department, Orthopedic Surgery Research Center (OSRC), Sina University Hospital Tehran University of Medical Sciences Tehran Iran

**Keywords:** fracture pattern complexity, illicit drugs, opium, tibial fractures, trauma

## Abstract

**Purpose:**

Illicit drug abuse is a global epidemic afflicting millions worldwide. Several studies have investigated the contribution of this dependence as a risk factor for fracture, but its impacts on fracture severity have been rarely studied. The present study primarily aims to determine the relationship between illicit drug abuse and the severity of tibial shaft fractures.

**Methods:**

This retrospective study consecutively included patients aged ≥18 years with tibial shaft fracture who attended Sina Tertiary Hospital, Tehran, Iran, between 2016 and 2021. The fracture patterns were assessed according to the Arbeitsgemeinschaft für Osteosynthesefragen Foundation/Orthopaedic Trauma Association classification. Participants were divided into three individual specialists into groups: simple (A), wedge (B) and multifragmentary (C) fractures. The association of illicit drug abuse and other recorded variables, including age, sex, body mass index (BMI), comorbidities, physical activity, smoking habits and mechanism of injury, was also examined and assessed in multivariate logistic regression.

**Results:**

Of 219 patients, 26 were drug abusers, and 193 had no history of use. A total of 20 out of 26 drug abusers experienced a complex fracture, yielding a rate of 76.9%, while this rate for nonusers was 50.3% (97 out of 193), indicating a statistically significant difference between the two subgroups (*p* = 0.011). The smoking history also influenced the fracture pattern (*p* = 0.027) based on univariate analysis; however, using adjusted multivariate analysis yielded only illicit drug abuse (odds ratio = 3.495; confidence interval = 1.144–10.680) as a risk factor for more complex fractures.

**Conclusion:**

The evidence from this study suggests that complexity and fracture patterns can depend on illicit drug abuse history.

**Level of Evidence:**

Level III.

**Abbreviations**
AO/OTA: Arbeitsgemeinschaft für Osteosynthesefragen Foundation/Orthopaedic Trauma Association
BMI: body mass index
CCI: Charlson comorbidity index
CI: confidence Interval
OR: odds ratio

## BACKGROUND

Tibial shaft fracture, as one of the most common fractures among long bones, imposes a significant cost to the healthcare systems. Additionally, it can lead to a decrease in the quality of life of patients and an increase in disability‐adjusted life years [1, 2]. Iranian reports introduced road traffic accidents as the most common cause of tibial fracture [3]. However, in some other studies, indoor activities, sports and walking were the major settings of trauma [4].

Being familiar with the risk factors affecting the various characteristics of this fracture plays an essential role in the design of treatment plans. A number of researchers have reported an association between fracture risk and opioid use, such as methadone or fentanyl, followed by a higher risk of falls due to dizziness [5]. Previous studies have also reported opioids and illicit drugs associated with a higher risk of nonunion bone fracture [6, 7]. The effect of opioids as a nonunion risk is of concern because there is an epidemic of opioid abuse in Iran and the United States [8, 9]. Opioid use has been found to influence longer hospital length of stay, worsening the outcome and greater costs and risk of readmission [10, 11].

However, much of the research up to now has focused on smoking, sex, age and underlying disease as factors impaction on tibial fracture healing [12, 13]. To the best of our knowledge, no previous study has investigated the opioid and illicit drug abuse relationship with the tibial shaft fracture pattern complexity; therefore, its possible effects remain unknown. This study aims to put the idea that “illicit drug abusers experience a more severe and complex tibial shaft fracture” to the test.

## METHODS

**Figure 1:**
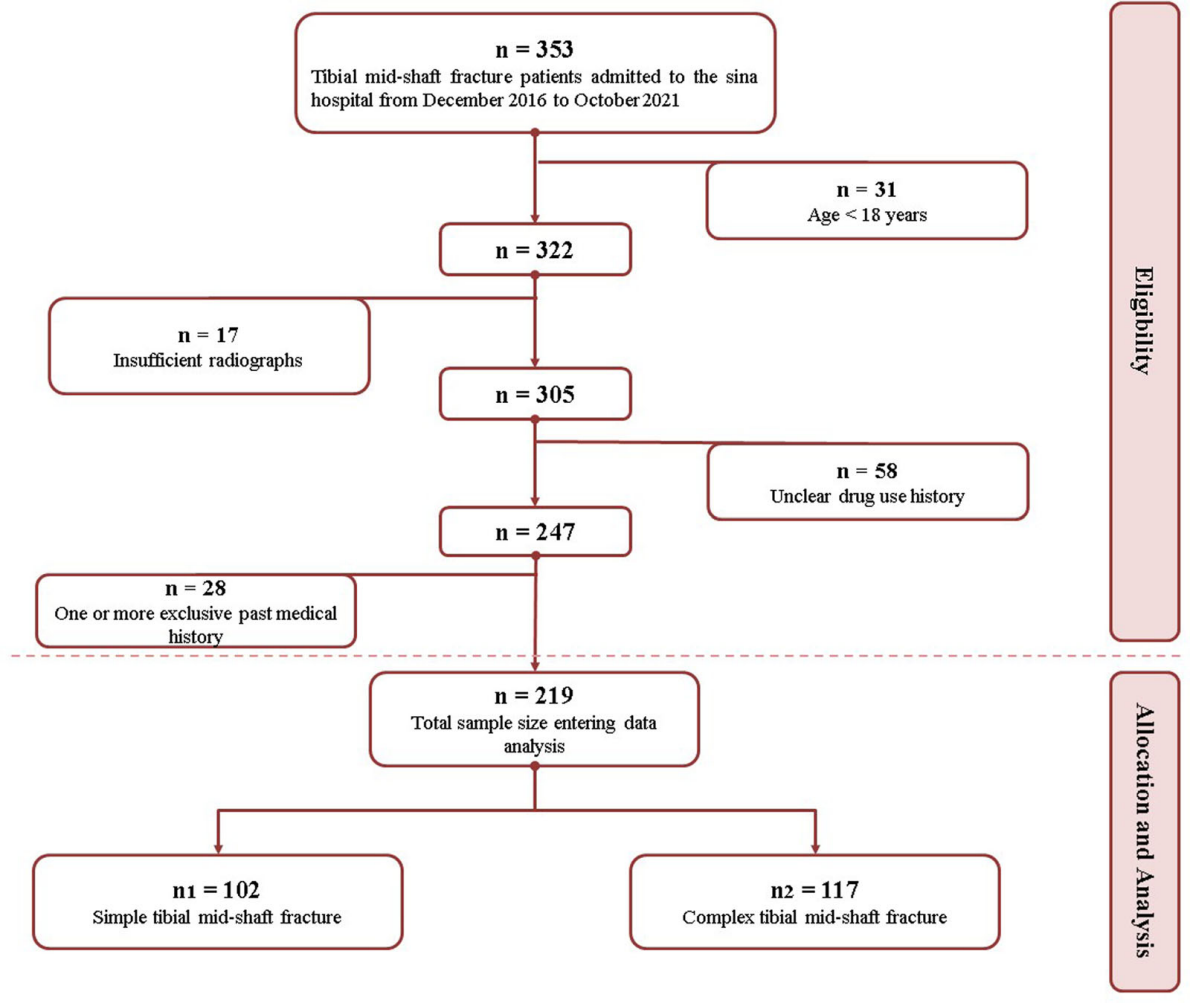
Patient's select graph.

This retrospective study was conducted on tibial shaft fracture patients who were admitted to our centre, from December 2016 to October 2021. Data were collected from the medical records, and missing data was completed via telephone calls. All participants with tibial shaft fractures, who were ≤18 years old, were excluded. Other exclusion criteria were: not having a plain radiographic image, lack of medical records data, lack of access to the patients via phone call (each patient lacking major data was called at least three times before exclusion), not having consent for collaborating, any previous tibial fracture, history of bone metastasis and related cancers and osteomyelitis (Figure 1).

**Figure 2:**
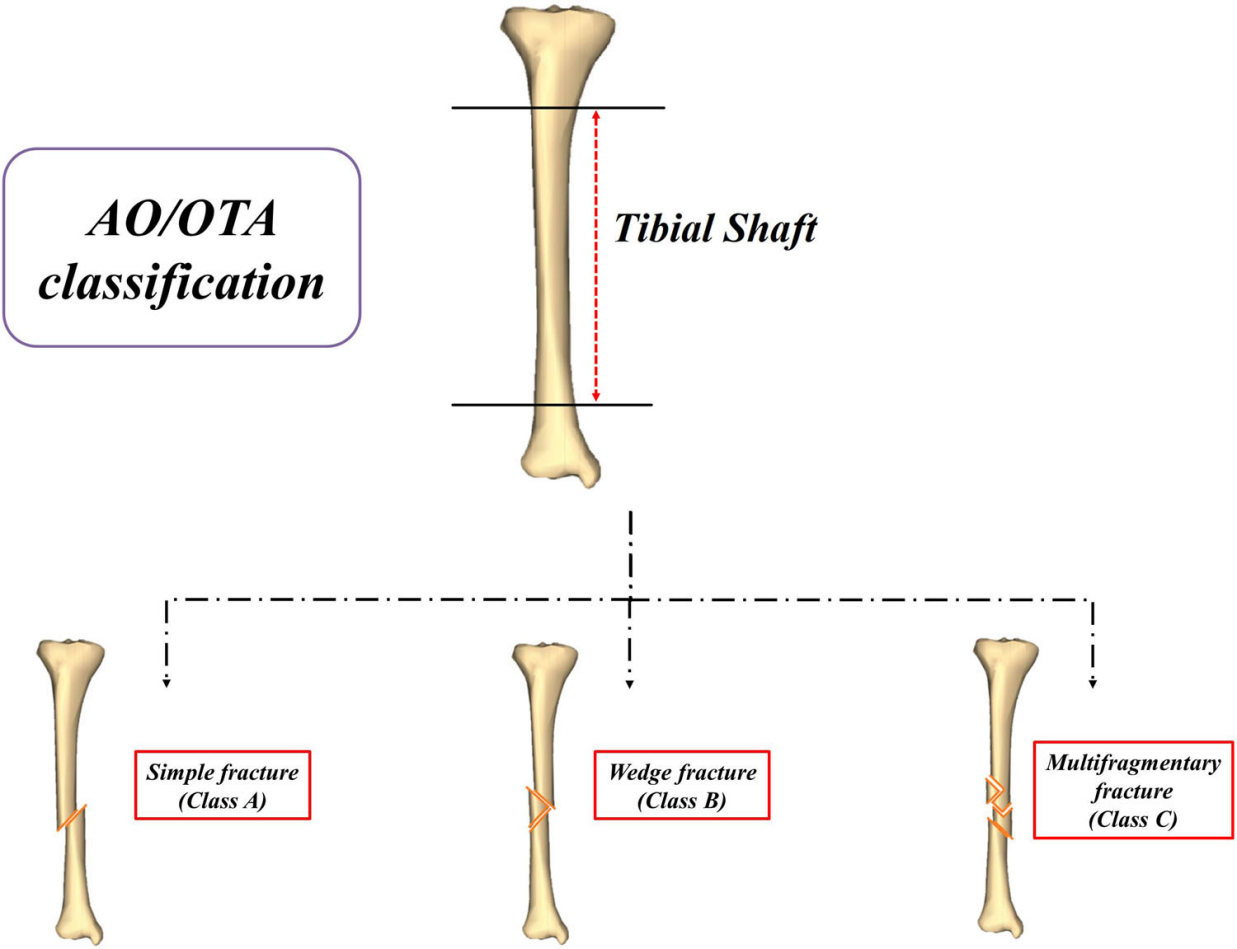
The Arbeitsgemeinschaft für Osteosynthesefragen Foundation/Orthopaedic Trauma Association (AO/OTA) classification of tibial midshaft fracture.

Patients were divided into three groups based on their plain radiographs and the 2018 revision of Arbeitsgemeinschaft für Osteosynthesefragen Foundation/Orthopaedic Trauma Association (AO/OTA) classification by two individual specialists, one orthopaedic surgeon and one radiologist (Figure 2) [14, 15]. In the disagreement, a third independent specialist made the final decision. Patients were required to have at least one anterior–posterior and one lateral view radiograph. In 181 participants, an agreement was achieved between the two investigators, and the third specialist assessed the remaining 38 fractures. The outcome of this study was defined as the complexity of tibial shaft fracture, thus cases were recruited from the wedge (group B) and multifragmentary fractures (group C), and the controls were ascertained from simple fractures (group A).

Exposure is identified as a history of illicit drug abuse [16], according to the Diagnostic and Statistical Manual of Mental Disorders, 4th edition, revised index. All variables consist of age, sex, body mass index (BMI), marital status (married or never married), exercise history (having physical activity for 30 min a day and at least 3 days a week or equivalent), current smoking, alcohol consumption (by asking a yes or no question: “Have you consumed alcoholic drinks more than one time during a lifetime?”), current calcium supplementation, current vitamin D supplementation, glucocorticoid (GC) current use, current bisphosphonate use, current oestrogen use, open or close fracture, high or low energy mechanism (high energy trauma is defined as fractures due to a traffic accident at more than 30 km/h or a fall from >3 m), polytrauma (more than one simultaneous fracture), mechanism of fracture (road accident or not), Charlson comorbidity index (CCI) (including inflammatory diseases, rheumatoid arthritis, hypothyroidism, hyperthyroidism, hypertension, diabetes mellitus, hyperparathyroidism, hypoparathyroidism, asthma and other endocrine and rheumatoid diseases), fracture pattern based on AO/OTA classification. These data were collected retrospectively from the medical records. In the case of missing data, needed information was collected via telephone calls. Each patient lacking crucial data was called at least three times before being excluded.

### Statistical analysis

Categorical variables were described as frequencies and continuous variables were described using mean and standard deviation (SD). Proportions for categorical variables were compared using the *χ* ^2^ test. An independent‐sample *t*‐test or a Mann–Whitney *U*‐test was used to compare the numeric variable. A logistic regression model was designed to calculate the adjusted odds ratios (ORs) with independent variables and fracture complexity as the dependent variable by the Windows OS version of SPSS v.26. A *p* value less than 0.05 was considered statistically significant. Significant variables from univariate analysis and possible confounding variables of fracture complexity based on previous studies were included in a multivariate model. Alcohol consumption [17], GC use [18], high energy trauma [19], age and sex [20] were identified by prior research as risk factors for pattern complexity of the fracture.

## RESULTS

### Patients characteristics

Of 353 patients screened at the beginning of this study, a total of 219 patients with tibial shaft fractures who met the inclusion and exclusion criteria entered the study. Among the study population, 91.3% (*n* = 200) were male, and the mean age was 40.04 ± 15.2 SD. It was revealed that 11.9% (*n* = 26), 51.1% (*n* = 112) and 11.4% (*n* = 25) of patients were illicit drug abusers, current smokers and alcohol consumers, respectively (Table 1).

**Table 1:** Patient characteristics (*n* = 219).

Variables	Illicit drug abusers (*n* = 26)	Overall frequency (%)
Sex (male)	25/26 (96.15)	200/219 (91.32)
Age (year) ± SD	39.62 ± 12.13	40.05 ± 15.20
BMI (*n* = 109) (kg/cm^2^) ± SD	24.42 ± 5.83 (*n* = 11^a^)	25.55 ± 4.71
Marital status (never married)	12/26 (46.15)	79/215^a^ (36.74)
Exercise	9/26 (34.62)	66/219 (30.13)
Current smoking	23/26 (88.46)	112/219 (51.14)
Alcohol consumption	4/26 (15.38)	25/219 (11.42)
Illicit drug abuse	—	26/219 (11.87)
Current calcium supplementation	2/26 (7.69)	28/219 (12.79)
Current vitamin D supplementation	4/26 (15.38)	32/219 (14.61)
GC current use	1/26 (3.85)	4/219 (1.83)
Bisphosphonate current use	0/26	6/219 (2.74)
Oestrogen current use	0/26	4/219 (1.83)
Open fracture	6/25^a^ (25)	62/207^a^ (29.95)
High energy	25/26 (96.15)	201/215^a^ (93.49)
Polytrauma	5/22^a^ (22.73)	65/202^a^ (32.18)
Mechanism of fracture (road accident)	20/26 (76.92)	161/215^a^ (74.88)
Charlson comorbidity index (*n* = 219)		
0–1	21/26 (80.77)	177/219 (80.82)
2–3	4/26 (15.38)	32/219 (14.61)
4–5	0/26	7/219 (3.20)
≥6	1/26 (3.85)	3/219 (1.37)

Abbreviations: BMI, body mass index; GC, glucocorticoids.

^a^ The number differs due to missing values.

Illicit drug abuse comprised 12 opioids, four methamphetamine, three methamphetamine plus opioids, two methamphetamine plus crack cocaine, two cannabis, one amphetamine, one phencyclidine and one cannabis plus opioids. Based on the AO/OTA classification, using the graphs taken at the time of admission, patients were classified into three subtypes: 102 (46.6%) simple (A); 79 (36.1%) wedge (B); and 38 (17.3%) multifragmentary (C) fractures. Although with more severe involvement, the mean age of the fracture subtype increased, this difference was not statistically significant (*p* = 0.075) (Table 2). Overall, no significant difference was seen between the three subtypes except for current smoking and illicit drug abuse prevalence, with *p* = 0.045 and 0.036, respectively (Table 2).

**Table 2:** Univariate associations with type A, B and C tibial shaft fractures according to the AO/OTA classification.

	AO fracture group			*p* Value	
Variables	A (*n* = 102)	B (*n* = 79)	C (*n* = 38)	A vs. B vs. C	A vs. B+C
Sex (male)	93/102	69/79	38/38	0.074	0.942
Age (year) ± SD	38.84 ± 15.85	39.15 ± 14.65	45.13 ± 13.85	0.075	0.355
BMI (*n* = 109) (kg/cm^2^) ± SD	25.67 ± 5.12	32.77 ± 4.38	24.90 ± 4.30	0.377	0.278
Marital status (never married)	41/100^a^	29/78^a^	9/37^a^	0.198	0.227
Exercise	35/102	20/79	11/38	0.418	0.208
Current smoking	44/102	43/79	25/38	0.045δ	0.027δ
Alcohol consumption	12/102	9/79	4/38	0.979	0.879
Illicit drug abuse	6/102	13/79	7/38	0.036δ	0.011δ
Current calcium supplementation	12/102	9/79	7/38	0.518	0.673
Current vitamin D supplementation	14/102	12/79	6/38	0.938	0.729
GC current use	3/102	1/79	0/38	0.460	0.341
Bisphosphonate current use	3/102	2/79	1/38	0.985	1.000
Oestrogen current use	3/102	1/79	0/38	0.460	0.341
Open fracture	22/94^a^	30/77^a^	10/36^a^	0.083	0.061
High energy	94/98^a^	72/76^a^	35/37^a^	0.916	0.754
Polytrauma	28/96^a^	26/73^a^	11/33^a^	0.383	0.665
Mechanism of fracture (road accident)	77/98^a^	52/76^a^	32/37^a^	0.082	0.471
Charlson comorbidity index					
0–1	84/102	65/79	28/38	0.59	0.792
2–3	13/102	11/79	8/38		
4–5	4/102	1/79	2/38		
≥6	1/102	2/79	0/38		

*Note*: Continuous variables are presented as mean with standard deviation, and categorical variables as numbers.

Abbreviations: AO/OTA, Arbeitsgemeinschaft für Osteosynthesefragen Foundation/Orthopaedic Trauma Association; BMI, body mass index; GC, glucocorticoids.

^a^ The number differs due to missing values.

^δ^ *p* < 0.05 is considered significant.

### Associations with fracture complexity

In this study, 117 (53.4%) patients suffered from complex fractures. The univariate analysis suggested alcohol consumption (*p* = 0.027) and illicit drug abuse (*p* = 0.011) as significant risk factors for fracture complexity. Age, sex, BMI, marital status, fracture mechanism, GC intake, cigarette smoking, opium abuse and calcium and vitamin D supplementation did not affect fracture complexity (Table 2). It is worth mentioning that being open or high energy was also not predictive of a more complex fracture (*p* = 0.061 and 0.754, respectively). Taking into account comorbidities through the CCI yielded no meaningful difference between simple and complex fractures (*p* = 0.792). When tested in logistic regression analysis, illicit drug abuse (odds ratio [OR] = 3.495, confidence interval [CI] = 1.144–10.680), in contrast to smoking history (OR = 1.720, CI = 0.860–3.440), remained associated with fracture complexity. This result demonstrates that a history of illicit drug abuse might be correlated with a more complex tibial shaft fracture (Table 3).

**Table 3:** Potential associates with intra‐articular distal radius fracture assessed in multivariate logistic regression analyses.

Exposure	Crude odds ratio (95% CI)	Adjusted odds ratio (95% CI)	*p* Value for adjusted
Sex (male)	0.966 (0.376–2.478)	1.008 (0.333–3.054)	0.989
Age (year)	1.010 (0.992–1.028)	1.011 (0.991–1.031)	0.285
Current smoking	1.829 (1.069–3.130)δ	1.632 (0.880–3.000)	0.115
Alcohol consumption	0.938 (0.407–2.158)	0.619 (0.315–1.990)	0.619
Illicit drug abuse	3.299 (1.270–8.573)δ	2.915 (1.070–7.940)δ	0.036δ
GC current use	0.284 (0.029–2.779)	0.251 (0.021–2.979)	0.273
High energy	0.759 (0.208–2.771)	0.702 (0.184–2.671)	0.604

*Note*: Continuous variables are presented as mean with standard deviation, and categorical variables as numbers.

Abbreviations: CI, confidence intervals; GC, glucocorticoids.

^δ^ *p* < 0.05 is considered significant.

## DISCUSSION

Interestingly, this study demonstrates that illicit abuse is strongly associated with the complexity of the tibial shaft fracture. Previous studies on traumatic orthopaedic patients have revealed that opioid users have greater odds of mechanical complications, adverse events, mortality, prolonged hospital stay, disability and psychological distress than nonopioid users [21, 22, 23]. Besides, it has also been demonstrated that opioid use itself might deteriorate bone density through opioid‐induced hypogonadism [24], leading to osteoporosis in young patients [22], which is associated with an increased risk of fracture and, subsequently, the complexity of fracture [25]. Regidor et al. found that the rate of injury, including assaults, burns and accidental poisoning, in heroin users was higher than in the control group [26].

In the meantime, some studies have found no association between cocaine use and mortality [27, 28, 29]. Alcohol or illicit use has adverse effects on the central nervous system causing depression, cognitive impairment, altered senses and judgement, confusion or delirium [30]. Subsequently, these effects put patients at high risk of being involved in violent acts and fatal accidents, leading to traumatic incidents. As a result of these behaviours, the prevalence of alcohol and illicit use in traumatic patients is reported to be high [31]. Physiologic derangements from alcohol and illicit drug use might worsen outcomes in traumatic patients [32].

On the other hand, a study by Daughters et al. showed that alcohol use could have a protective effect on trauma [33]. Our results demonstrate that illicit use is associated with the fracture's complexity. When adjusting for confounding factors, a stronger correlation was found between illicit use and fracture complexity. In a retrospective study by Sokoya et al. [34], it was demonstrated that marijuana legalization does not affect the pattern of facial fracture.

We did not find any association between sex, marital status and complexity of the fracture similar to previous results in the literature [35]. However, our results regarding the relationship between sex and fracture complexity should be interpreted with caution as men comprised most of our study population and our data are heavily biased towards male participants.

Smoking was significant in univariate analysis however when taken into the logistic regression model it turned out to be insignificant. This might be due to the association of smoking with illicit drug abuse and the fact that most of the drug abusers in our study were also current smokers (Table 1). Previous studies' findings on the relationship of smoking with fracture complexity are heterogeneous. Hjelle et al. found current smoking a protective factor against complex fractures, while other studies found smoking to be insignificant in regard to fracture complexity [17, 18, 20]. However, it should be noted that all of these studies were conducted on patients with distal radius fracture, had a significantly older sample size and occurred mostly due to low energy mechanisms.

No association was observed between alcohol use and the complexity of the fracture. In addition, oestrogen, bisphosphonate, calcium supplementation and vitamin D supplementation did not appear to be associated with the fracture's complexity. Previous studies indicated no correlation between osteoporosis and AO classification of fractures [18, 36, 37, 38]. We did not observe any significant association between Charlson score and fracture complexity. Also, doing exercise was not associated with the complexity of the fracture, which was in accordance with a previous study [17].

Moreover, none of the high‐ or low‐energy trauma and fracture mechanisms were associated with fracture complexity. This is in contrast to a previous study that found low‐energy mechanisms to be protective for fracture severity [18]. We sought to address this discrepancy as the energy of trauma seems to be a fairly obvious factor influencing the complexity of fracture. We should consider that the retrospective nature of this study, recall bias, variety of definitions and missing data might have influenced this result. Another possible explanation is that the majority of our patients had been admitted due to a high‐energy incident, which makes it difficult to interpret this variable.

To the best of our knowledge, no other study has reported the factors that could influence the tibial fracture's complexity. There is still a shortage of evidence about this issue. Cognitive status and recall bias might have influenced the results. The findings should be interpreted in light of several potential limitations. First, patients self‐reported their drug use, and some patients might have avoided accurate drug use reports due to moral, religious and legal issues. Second, we did not consider the type and quantity of drugs used by patients. However, it is almost impossible to obtain the frequency and dose of drug use due to differences in types, purity and administration routes. Third, the study's retrospective nature is subjected to several biases, and we cannot infer causal effects due to the lack of randomization. A prospective study of nonuser matched with the drug user groups could strengthen the results of our study. Fourth, because of the retrospective nature of our study and the fact that vitamin and mineral levels or bone mineral density are not routinely evaluated in trauma patients in our centre, we could only study the impact of vitamin and mineral supplementation and were unable to investigate the relationship between their blood levels and fracture complexity. We suggest that future studies should take into account this information. Finally, despite AO classification being one of the major classification systems, retesting the results of this study using other classification systems may clarify this subject even more.

## CONCLUSION

In summary, the results of this study demonstrate that patients with shaft tibia fractures who abused illicit drugs experienced higher odds of developing a complex fracture. Regarding alcohol, no association of its use with the fracture's complexity was documented. Level one preventive measures to identify illicit drug users and enlighten them could prevent developing complex fractures that put the economic, societal and clinical burden on the patient and the healthcare system.

## AUTHOR CONTRIBUTIONS

**Amirmohammad Sharafi**: Study design; data gathering; writing manuscript. **Ali Ghaderi**: Data gathering; writing manuscript. **Parmida Shahbazi**: Data gathering; writing manuscript. **Amirhossein Ghaseminejad‐Raeini**: Data gathering; writing manuscript. **Akam Ramezani**: Data gathering. **Mohammad Soleimani**: Manuscript reviewing; data analysis. **Parham Talebiyan**: Manuscript reviewing. **Seyyed Hossein Shafiei**: Study design; final supervision; manuscript reviewing.

## CONFLICT OF INTEREST STATEMENT

The authors declare no conflict of interest.

## ETHICS STATEMENT

This study was approved under the code: IR.TUMS.SINAHOPITAL.REC.1400.089 by the institutional ethics committee. All participants were contacted for their consent, and if not interested, they were excluded from the study. There is complete consent from the authors of the article for the publication of the article.

## Data Availability

The data of this study is at the disposal of the authors and is available upon reasonable request.
